# Fusion of the 2nd with the 3rd Cervical Vertebrae (C2-C3): A Case Series with Possible Clinical Significance

**DOI:** 10.1155/2023/3577693

**Published:** 2023-12-01

**Authors:** Eirini Demeneopoulou, Dorina Papa, Ilias Giotas, Angelos Nikolaou, George Tsakotos, Vasilios Karampelias, Theodore Mariolis-Sapsakos, Dimitrios Filippou, Maria Piagkou

**Affiliations:** ^1^Department of Anatomy, Medical School, National and Kapodistrian University of Athens, Greece; ^2^Department of Anatomy and Histology, Nursing School, National and Kapodistrian University of Athens, Greece

## Abstract

**Purpose:**

The current case series describes three cases of fusion between the 2nd cervical vertebra, the axis (C2), and the 3rd cervical vertebra (C3), creating a C2-C3 osseous complex and highlighting its morphological type of fusion (partial or complete) and morphometric details. The developmental background of this complex is emphasized, pointing out the possible clinical significance.

**Materials and Methods:**

The osseous complexes were derived from disarticulated skeletons of body donors and were collected from the osseous collection of the Anatomy Department of the Medical School of the National and Kapodistrian University of Athens.

**Results:**

Three blocked vertebral complexes (2 partial and 1 complete C2-C3 osseous masses) were identified. In two cases, the vertebral bodies were partially fused and in one case were completely fused. In the 1st case, the C2-C3 complex had fused spinous processes and distinct transverse processes. Facets were completely fused on the left and partially fused on the right side. In the 2nd case, the C2-C3 complex had partially fused vertebral bodies and distinguishable spinous processes. In the 3rd case, the C2-C3 complex had completely fused vertebral bodies, facets, laminae, and transverse and spinous processes.

**Conclusions:**

Among the three (C2-C3) fused osseous complexes, the two were partially and the one was completely ossified. The fused vertebrae were characterized by osteophytic formations (at the dens and C3 area) and osteoporotic lesions. Taking into consideration the C2-C3 fusion, and possible coexisted variants, particular caution should be made in the upper cervical area, to interpret possible neurological manifestations and to reach a safe surgical plan.

## 1. Introduction

A clinically important morphological variant of the cervical spine is the complete or partial fusion (synostosis) of two or more cervical vertebrae (CV). The fused or blocked vertebrae function as one. The most frequent area of fusion occurs at the level of the 2nd with the 3rd CV, thus creating the C2-C3 osseous complex, with a prevalence varying between 0.10% and 1.33%, followed by the fusion of the 5th with the 6th CV [1, 2]. The CV congenital fusion results after malformations of the notochord, associated with defects of the cervical somites, while the acquired fusion is often associated with trauma, infections, and juvenile rheumatoid arthritis [3]. The CV congenital fusion, usually asymptomatic until adulthood, is the main characteristic of the Klippel-Feil syndrome (KFS), usually presented with a short neck, limited neck motion, and a low posterior hairline [4]. The commonest symptoms include pain and neurologic manifestations, such as numbness, and a decreased range of cervical motion [5]. The degenerative changes may cause symptoms due to compression on cervical nerve roots [6] or may cause instability of the hypermobile articulations adjacent to the area of synostosis [4, 7]. Detailed knowledge of the occurrence of the fused cervical vertebrae is critical during upper cervical spine surgery and anesthetic procedures.

The current report performed on dried vertebrae highlights three unusual cases of fusion between C2 and C3 vertebrae, highlighting their morphology (type of fusion, complete or partial) and providing further morphometric details and possible clinical impact.

## 2. Case Presentation

**Figure 1:**
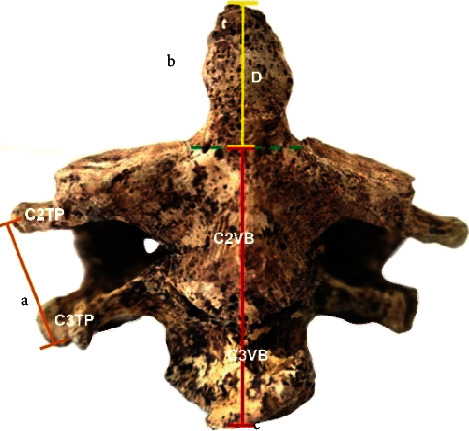
The measured distances of the fused cervical vertebrae C2-C3: a—distance between the transverse processes (TP) of the fused cervical vertebrae (C2TP-C3TP), b—the odontoid process (dens-D) height (yellow line, calculated from the base of the dens to its tip), and c—the height of the fused cervical vertebral bodies (red line—C2VB-C3VB).

**Figure 2:**
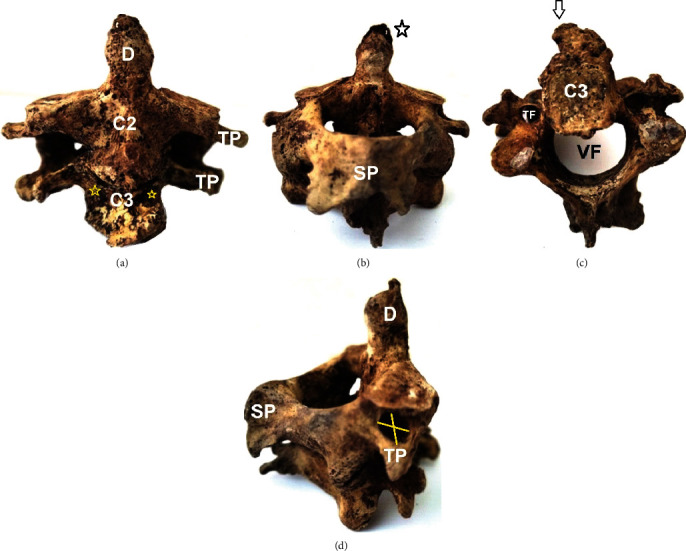
1st case of the fused vertebrae C2-C3: (a) anterior view, a small cleft between inferior and superior margins of the vertebral bodies (yellow asterisks), TP: transverse process; (b) posterior view, dens-D (black asterisk), SP: spinous process; (c) inferior view, C3 vertebra anteroinferior osteophyte formation (black arrow), TF: transverse foramen and VF: vertebral foramen; (d) right lateral view, TP anteroposterior and laterolateral maximum diameters (yellow lines).

**Figure 3:**
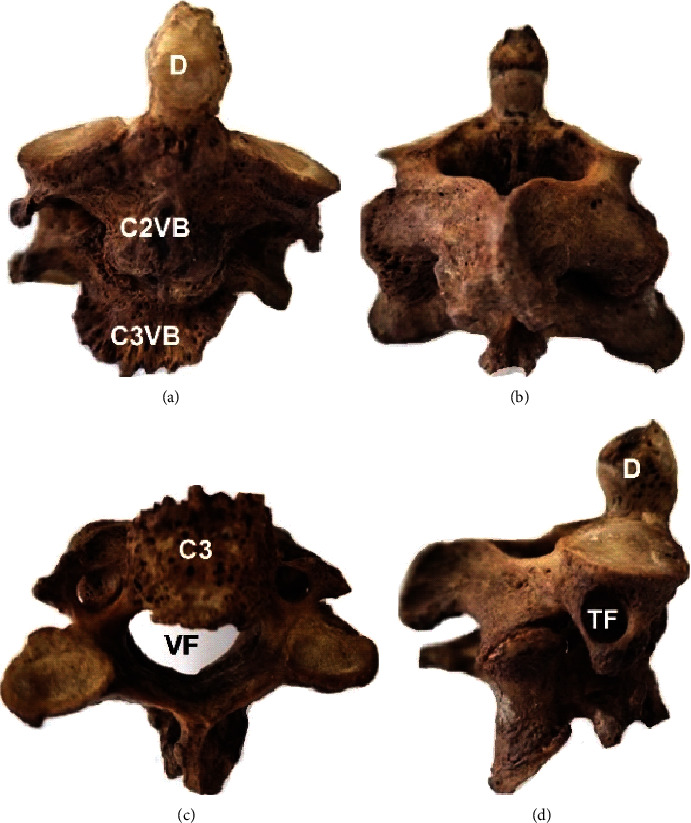
2nd case of the fused vertebrae C2-C3: (a) anterior view of the vertebral bodies C2 and C3 (C2VB, C3VB), D-dens; (b) posterior view; (c) inferior view, C3 vertebra, VF: vertebral foramen; (d) right lateral view, TF: transverse foramen.

**Figure 4:**
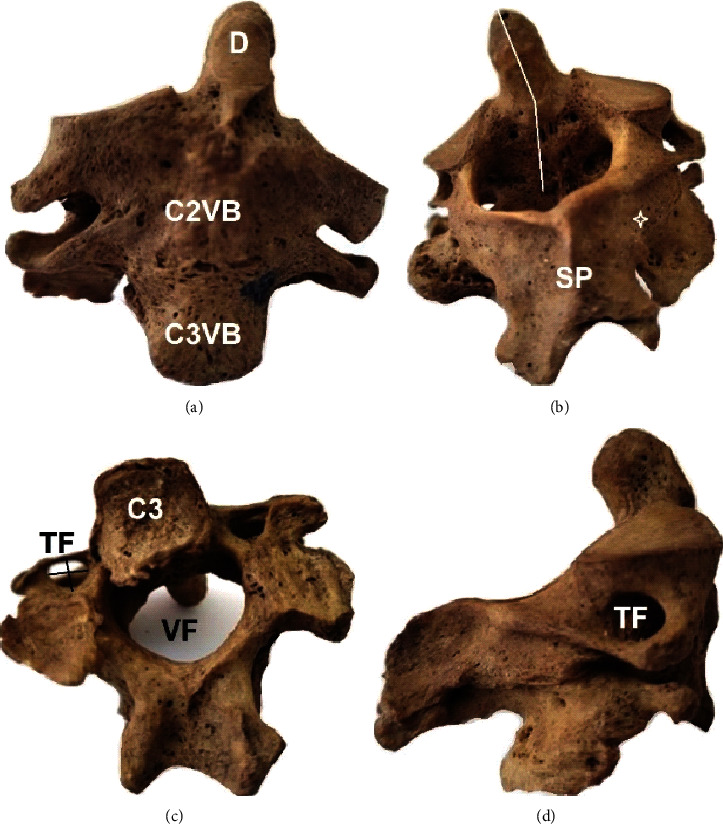
3rd case of the fused vertebrae C2-C3: (a) anterior view of the vertebral bodies of C2 and C3 (C2VB and C3VB), D-dens; (b) posterior view, in which the inclination of the fused complex (white line) is depicted, SP: spinous processes fused; white asterisk: the fusion at the area of the pedicles; (c) inferior view of the C3, VF: vertebral foramen, TF: transverse foramen diameters in black color; (d) right lateral view, TF: transverse process.

Three cases of fused vertebrae were identified at the C2-C3 level. The dried bones belonging to disarticulated skeletons of body donors of Greek origin were part of the osseous collection of the Anatomy Department of the Medical School of the National and Kapodistrian University of Athens. In all dried fused complexes, the morphology of the fused vertebral complexes was recorded by identifying the type of fusion (complete or partial) between vertebral bodies, pedicles, facets, laminae, and transverse and spinous processes. The morphometry of the fused CV was calculated by measuring in millimeter; the following are the distances (using a digital sliding caliper, Mitutoyo, accuracy 0.01 mm): (1) the intertransverse distance, i.e., distance between the transverse processes of the C2-C3 complex (Figure 1 (a)), (2) the height of the odontoid process (dens) (Figure 1 (b)), (3) the height of the fused vertebral bodies (Figure 1 (c)), (4) the maximum anteroposterior and laterolateral (sagittal) diameters of the transverse foramina (Figure 2(d)), and (5) the anteroposterior and laterolateral (sagittal) diameters of the vertebral foramen (spinal canal) (Figure 2(e)).

### 2.1. C2-C3 Morphology

In all cases, the vertebral bodies of the C2-C3 complex were fused (in 2 cases partially and in one completely). In the 1st case, the fused complex of the male body donor of unknown age at death had fused spinous processes and distinct transverse processes. Facets were completely fused at the left and partially fused at the right side (Figure 2). The lower border of the C3 vertebral body had a characteristic osteophytic formation (Figure 2(c)). In the 2nd case, the fused complex of the female body donor of unknown age at death had osteoporotic lesions and distinguishable spinous processes. A cleft was identified, at the right quadrant of the intervertebral disk (due to the partial fusion of the vertebral bodies) (Figure 3). In the 3rd case, the fused complex belonged to a 54-year-old male with tuberculosis and was characterized by a completely fused vertebral bodies, facets, laminae, and transverse and spinous processes. The C2-C3 complex is inclined to the vertical axis to the left side (Figure 4).

### 2.2. C2-C3 Morphometry

In the first case, the C2-C3 complex had a height of 38 mm, and the dens' height was 19 mm. The anteroposterior and laterolateral diameters of the vertebral foramina were 19 mm and 21 mm (C2 superior border) and 14 mm and 21.2 mm (C3 inferior border). The distance between the transverse processes (intertransverse distance) was 23 mm on the left and 21 mm on the right side. The maximum anteroposterior and laterolateral diameters of the transverse foramina in the C2 level were 4.91 mm and 6.01 mm on the right and 3.82 mm and 5.45 mm on the left side, respectively. In the C3 level, the maximum anteroposterior and laterolateral diameters of the transverse foramina were 7.2 mm and 9.21 mm on the left and 6.12 mm and 8.78 mm on the right side, respectively. In the 2nd case, the C2-C3 complex had a 42 mm height and the dens' height was 14 mm. The anteroposterior and laterolateral diameters of the vertebral foramina were 20 mm and 21.3 mm (C2 superior border) and 11 mm and 20 mm (C3 inferior border). The intertransverse distance was 15 mm on the right and 12 mm on the left side. The maximum anteroposterior and laterolateral diameters of the transverse foramina in the C2 level were 6.2 mm and 9.01 mm on the left and 6.5 mm and 9.32 mm on the right side and in the C3 level 5.9 mm and 8.97 mm on the left and 5.3 mm and 8.23 mm on the right side. In the third case, the C2-C3 osseous complex had a height of 36 mm, and the dens' height was 16 mm. The anteroposterior and laterolateral diameters of the vertebral foramina were 23 mm and 22.1 mm (C2 superior border) and 18 mm and 21.7 mm (C3 inferior border). The intertransverse distance was 11 mm on the left and 9 mm on the right side. The maximum anteroposterior and laterolateral diameters of the transverse foramina, at the C2 level, were 5.2 mm on the left and 5.1 mm on the right side, and at the C3 level, it was 4.7 mm on the left and 4.3 mm on the right side, respectively.

## 3. Discussion

### 3.1. Developmental Anatomy and Genetics

Congenital synostosis is the result of complex mechanisms during embryonic development. These anomalies are speculated to result from failure of segmentation of the cervical somites during the 3rd to 8th gestational week [8]. The chorda dorsalis fails to form the nucleus pulposus, resulting in a rudimentary fibrous intervertebral junction or the complete absence of any disk-like structure. The responsible gene is speculated to be Pax-1, which contributes to the fetal spine development [7]. It is also suggested that there might be a disturbance of the normal spinal subdivision due to a decrease in the blood supply [9]. Microtrauma during development may activate local factors, such as bone morphogenic proteins or prostaglandins that induce mesenchymal cells' modification to osteoblasts. As for the KFS, a rare congenital skeletal disorder presented with short neck, limited neck mobility (due to fused CV), and low posterior hairline [10], and it has been linked with mutations in GDF6, GDF3, and MEOX1 genes. The products of the first two are essential for the formation of bones and joints, while the protein produced by the lateral has a significant role in separating vertebrae [11].

### 3.2. Background of the Fused Cervical Vertebrae

#### 3.2.1. Prevalence

Several authors in their studies among several populations recorded fused CV with a prevalence ranging from 0.10% to 6.25%, fused thoracic vertebrae with a prevalence ranging from 0.08% to 4.16%, and fused lumbar vertebrae with a prevalence ranging from 0% to 2.08% [1, 2, 12–14]. Soni et al. [15] identified fused vertebrae in decreasing order of frequency for C2-C3 and C5-C6 levels with no gender preference, while they recorded that up to 70% of the atlantooccipital fusion cases coexisted with C2-C3 fusion with atlantoaxial joint instability. Natsis et al. [16] in their study on the 1st CV occipitalization found one skull, with atlantooccipital fusion that coexisted with a C2-C3 fusion, a prevalence of 0.6%.

#### 3.2.2. Morphometry

In the current series, the sagittal (laterolateral) diameter of the spinal canal ranged from 21 mm to 22.1 mm (C2 superior border) and from 20 mm to 21.7 mm (C3 inferior border). Ulbrich et al. [17] recorded spinal canal laterolateral diameters at the C1 level ranging from 10.7 to 19.7 mm and at the C3 level ranging from 9.4 to 17.2 mm. Normally, the variability of these dimensions is associated with the spinal level, gender, age, and height of the investigated subjects. Congenital fused CV are characterized by a decrease in the sagittal (laterolateral) diameter of the vertebrae [18]. In the current series, the C2-C3 complex had a height ranging from 36 mm to 42 mm. The height of the dens ranged from 14 mm to 19 mm. The anteroposterior diameter of the C2-C3 complex ranged from 19 to 23 mm (at the C2 superior border) and from 11 to 18 mm (at the C3 inferior border). Vadgaonkar et al. [19] recorded the mean anteroposterior diameter of the normal axis at 46 ± 0.5 mm and of the C3 vertebra at 47 ± 0.8 mm. The used C2-C3 complex had an anteroposterior diameter of 44 ± 0.2 mm. The body length of fused C2-C3 was 36 ± 0.8 mm.

### 3.3. Pathological Background of the Fused Vertebrae and the Modified Morphology

#### 3.3.1. The Klippel-Feil Syndrome (KFS)

According to Smith and Griffin [8], the prevalence of KFS is 0.6%. The C2-C3 fusion was the most common in those patients, with an incidence of 72.7% [5]. KFS clinical features consist of the triad, low posterior hairline, short neck, and decreased range of motion, which is observed in 50% of the patients [8]. In cases of asymmetrical fused CV, torticollis can develop with a markedly titled position of the head [20]. Fused vertebrae may also be identified in a rare autosomal recessive congenital anomaly, the spondylocarpotarsal syndrome [21]. Another rare autosomal dominant condition, the Wiedemann-Steiner syndrome, is characterized by abnormal development of the cervical spine (including fused CV, C1 and C2 abnormalities, small foramen magnum, and Chiari malformation type I) [22]. In a severe developmental craniovertebral junction abnormality with head retroflection, the so-called iniencephaly, the CV marked lordosis coexists with a cervical vertebra duplication, an irregular form of fused CV, a widened foramen magnum, and a small posterior cranial fossa [23]. Lopez-Espina et al. [24] emphasized the relationship between multilevel cervical fusion and its effects on disc degeneration (a 96% increase in the annulus nucleus and endplates) and osteophyte formation. They also recorded a higher stress in cases of double than a single fusion. Sonnesen [25] pointed out that the fusion of the cervical vertebral column is associated with the development and function of the craniofacial morphology in the sagittal, vertical, and transversal planes. An association was identified by Sonnesen et al. [26] between the fusion of cervical vertebral bodies and head and neck posture.

#### 3.3.2. Clinical Signs and Symptoms

Fused CV may be asymptomatic until adulthood and are often identified incidentally, in imaging [4]. In later stages, the degenerative changes in the cervical spine and the osteophytes' presence and intervertebral disc tears in adjacent regions may cause nerve compression and symptoms' presentation [6]. In congenital fused CV, alterations occur in biomechanics, resulting in extra stress on adjacent segments, progressive joint degeneration, spinal canal stenosis, and segmental instability [4, 7]. A narrowed intervertebral foramen may also cause nerve compression, leading to sensory and motor abnormalities. Subjects with C2-C3 fusion often had symptoms associated with dens dysplasia and occipitocervical instability. Degenerative changes at the unfused segment and a narrow bony canal are high-risk factors in the development of neurological compromise [27]. In KFS, the commonest symptoms that may lead to diagnosis are pain, neurologic symptoms, and decreased cervical range of motion [5]. Reported symptoms include neck, upper extremity, or cervical axial pain, numbness, tickling, bilateral upper extremity weakness, ataxia, spasticity, headaches, and muscle atrophy [28]. KFS also predisposes to severe neurologic injury following minor trauma, such as spinal cord injury, facet dislocations, or even death. It is critical for patients with degenerative changes in the cervical vertebral column to be aware and protect themselves, against even mild possible trauma, avoiding extensive rotation and extension of the head that could induce spinal cord or vertebral artery compression [15]. The suggestion against a potential injury is to avoid contact sports.

#### 3.3.3. Surgical Implications

Fused CV may complicate neurosurgical and anesthetic procedures, due to the altered anatomy and reduced mobility [8]. Neck hyperextension during endotracheal intubation can predispose to intervertebral disc collapse in patients with fused CV. Thus, knowledge of the upper cervical fusion and its topographic relations is of paramount importance for surgeons when interpreting imaging studies to plan a safe and successful surgery [16, 29].

### 3.4. Study Limitations

The current case series has the following limitations: The unknown age of the subjects and the probable pathological background (lack of details from the subjects' medical records) did not permit us to discuss further the possible association of C2-C3 fusion with the pathology. The lack of morphometric details, concerning the pedicles of the vertebrae, and their variant morphology were omitted. Thus, no emphasis was given to the surgical planning for these types of deformities [29]. A major limitation is the description of monosegmental specimens without considering their relationship to the rest of the cervical spine. Other limitations were the absence of the related skulls which makes impossible the association of the fused CV with the atlantooccipital fusion. The lack of information concerning the adjacent tissues did not permit us to give our series a further clinical application.

## 4. Conclusions

Three cases of C2-C3 fusion (two partial and one complete) were identified. The morphology (partial or complete fusion) and morphometry of the C2-C3 fused complexes are representative of the clinical manifestations. The fused CV except for the alterations in morphology and morphometry may coexist with osteophytic formations (dens and C3 area) and osteoporotic lesions. Taking into consideration the fusion at the C2-C3 level, a possible atlantooccipital fusion and other variants (C1 and C2 abnormalities, foramen magnum or spinal canal stenosis, and occipitocervical instability) may also coexist that further should be identified in patients in order to interpret possible neurological manifestations, as well as to reach a safe surgical plan, when approaching in this area.

## Data Availability

The data used are included in this article.
